# London Fever Hospital

**Published:** 1902-05-03

**Authors:** 


### THE INSTITUTIONAL WORKSHOP.

### LONDON FEVER HOSPITAL.

### CENTENARY COMMEMORATION AT THE MANSION HOUSE.

ON Monday afternoon, April 28th, the fact that the year 1902 marked the completion of 100 years' work of this hospital was celebrated in a very practical way by some hundreds of people who responded to the invitation of the Lord Mayor and the Lady Mayoress at the Mansion House. At 4.30 tea and coffee were provided, and the Lawler Quintette gave a selection of music in the anteroom, and then the guests poured into the large Egyptian Hall, where they awaited the arrival of their host and hostess, with the Princess Louise, Duchess of Argyll, and His Grace the Duke of Argyll. By the time the distinguished company arrived people were overflowing into the anteroom, and standing several deep round the sides of the Hall.

The Lord Mayor, who was in semi-state, said he was quite sure that the first words those present would wish him to say were words of thanks to Her Royal Highness for coming. "We see," he said, "every hour in the Royal Family the wish to further the ends of charity." It was seen, he continued, in double measure in Queen Victoria, of ever blessed memory; she was always deeply interested in this institution, and His Majesty the King now held the position of President. He hoped the appreciation of their continued interest would be shown in a practical way, for the charity which had been in existence just a century had done good solid work in that time for the welfare of humanity.

The Lord Mayor then slightly sketched the history of the foundation of the hospital, 100 years ago, and concluded with an eloquent appeal to the public for funds to enable it to carry on its work and to undertake the necessary rebuilding. As treasurer, he felt amazed sometimes that the public did not appreciate the hospital more fully. Not until a family was brought face to face with the necessity of isolation did they realise its value, and then the promptitude with which the infected person was removed to the hospital brought the facts home to them.

Even though they might not have fever in the house, they ought to support this hospital, if only as a kind of insurance. His business, he concluded, was to introduce one who could tell much more about the hospital than he could, one who himself took a keen interest in it, and whose father had been treasurer before him.

The Duke of Argyll said he could only speak as an amateur, since the Lord Mayor had forestalled him in the remarks about the hospital which he intended to have made. He would remind them of the discovery of Sir William Jenner who was able, in the wards of the London Fever Hospital, to differentiate between typhus and typhoid fever. Since that time science had gone a long way towards fighting typhoid. The hospital had existed 100 years, and it seemed necessary in all these old hospitals to do a certain amount of rebuilding. They would be aware, from their own experience, that it was quite as expensive to repair and alter as to build an entirely new structure. Then there was a great need of a convalescent home. That had not been mentioned in the Lord Mayor's speech. It was most necessary for a hospital situated in a town. Lord Lister had told him some time ago, at a dinner given to draw public attention to a hospital for the study of malaria, that he considered that any case of diphtheria, taken in time, could be cured. Unfortunately, so far as enteric fever was concerned, there had been only too much field for study recently, as well as for experiment in inoculation, and he was sorry to say that the experiments had not, so far, come up to the expectations entertained. There was an interesting set of models at South Kensington Museum illustrating the history of a mosquito, and showing how the red corpuscles were attacked and gradually vitiated, and how the mosquito's fell purpose was effected. This showed how it had become possible to study the progress of infection. The ordinary flea conveyed infection something in the same way; it was the bearer of the plague bacillus. In the fur of the clean and healthy rat it was comparatively harmless, but when the rat was seedy and turned his face to the wall, the fleas left him and spread disease. He believed this had been found to be the case in Glasgow and Liverpool recently; he made the remark because it showed that it was only by such investigations that disease could be alleviated. He appealed for further help, in order to have this hospital reconstructed and thoroughly equipped. Isolated cases might be treated by the doctors very successfully, but such cases were not recorded as the cases in a large hospital were, and these were of very great value in the study of disease. "This great assemblage," the speaker concluded, "shows the interest taken in the hospital. It has been said by some that one should always take a little whisky with everything; I beg to improve on that axiom, and to suggest that the best specific against disease is to contribute a little money to the hospital."

The Lord Mayor said the time had come to show a practical interest in the hospital, and he would ask that the papers placed in everyone's hands should now be filled up. He hoped they would show the Princess that they were not ungrateful for the honour she had done them.

For some moments there was silence, and while the committee went round to collect contributions, Judge Lumley Smith proposed a vote of thanks to Her Royal Highness and His Grace the Duke of Argyll. This, he said, should have been done by their President, Lord Balfour of Burleigh, who, however, had just met with a slight accident, which prevented his being present. He wished to emphasise the fact that it was the middle classes, or perhaps the lower middle classes, for whom this hospital was of such importance. To the poor man illness came as an additional misery to which he resigned himself, but nothing could exceed the horror with which it was discovered that one member of a lower middle class family had contracted an infectious disease. They might picture the distress of the workgirl, or the clerk, on finding that it was impossible to go to work for fear of taking infection to the place of business; and it was this struggling class of society that suffered. His Grace the Duke of Argyll had alluded to the decline and fall of the fever bacillus; that was what they wanted to bring about. It was 60 years since a member of the Royal Family first took an interest in this hospital; the Prince Consort was the titular president for 40 years, and the present King had been patron for 20 years, while the late Queen Victoria was a liberal donor. He was quite sure that the interest of Her Royal Highness the Princess and of His Grace would continue.

Alderman and Sheriff Bell seconded the motion, which was carried unanimously and replied to by the Duke of Argyll, who said that the Princess had had practical experience of the value of the hospital in connection with her household.

The Princess then received the money collected in the room, and Major Christie, secretary of the hospital, read a long list of sums given before the meeting began; among other donors were the Bank of England, which gave £105, Mr. Cargullan had made two of his children life-governors by giving for them ten guineas each, and an anonymous donor had sent a contribution "on account of the hospital being a paying one." The Lord Mayor had given twenty-one guineas, and the Lady Mayoress ten guineas. Altogether, £92 17s. had been received in new subscriptions, and £1,398 9s. in donations. When the amount collected in the room was counted it was found that some £100 had been received in cash, while there were promises to the amount of another £100, making a total of £1,800.

Mr. Edward Norman then proposed a vote of thanks to the Lord Mayor and Lady Mayoress. The Lord Mayor, he said, was not only treasurer, but supported the hospital in every possible way; the Lady Mayoress was arranging an entertainment for May 12th, and their daughter, Mrs. Dent, had recently collected £18 for the funds. This was seconded by Mr. Stewart, who said the City was always forward in the cause of charity.

The Lord Mayor, in replying, said that the grand hall in which they were assembled had been the pivot round which charity had worked for many years, and he hoped that no Lord Mayor would be backward in keeping up the prestige of the Mansion House. This had been a most successful meeting, and its success was entirely due to the Princess. If there was one trait more than another that endeared the Royal Family—and might God's blessing be poured upon them—to the nation, it was their giving of themselves and their position to the furthering of charity.

